# Mechanical Stimulation Induces mTOR Signaling via an ERK-Independent Mechanism: Implications for a Direct Activation of mTOR by Phosphatidic Acid

**DOI:** 10.1371/journal.pone.0047258

**Published:** 2012-10-15

**Authors:** Jae Sung You, John W. Frey, Troy A. Hornberger

**Affiliations:** 1 Program in Cellular and Molecular Biology, University of Wisconsin- Madison, Madison, Wisconsin, United States of America; 2 Department of Comparative Biosciences in the School of Veterinary Medicine, University of Wisconsin - Madison, Madison, Wisconsin, United States of America; University of Texas Health Science Center at Houston, United States of America

## Abstract

Signaling by mTOR is a well-recognized component of the pathway through which mechanical signals regulate protein synthesis and muscle mass. However, the mechanisms involved in the mechanical regulation of mTOR signaling have not been defined. Nevertheless, recent studies suggest that a mechanically-induced increase in phosphatidic acid (PA) may be involved. There is also evidence which suggests that mechanical stimuli, and PA, utilize ERK to induce mTOR signaling. Hence, we reasoned that a mechanically-induced increase in PA might promote mTOR signaling via an ERK-dependent mechanism. To test this, we subjected mouse skeletal muscles to mechanical stimulation in the presence or absence of a MEK/ERK inhibitor, and then measured several commonly used markers of mTOR signaling. Transgenic mice expressing a rapamycin-resistant mutant of mTOR were also used to confirm the validity of these markers. The results demonstrated that mechanically-induced increases in p70^s6k^ T389 and 4E-BP1 S64 phosphorylation, and unexpectedly, a loss in total 4E-BP1, were fully mTOR-dependent signaling events. Furthermore, we determined that mechanical stimulation induced these mTOR-dependent events, and protein synthesis, through an ERK-independent mechanism. Similar to mechanical stimulation, exogenous PA also induced mTOR-dependent signaling via an ERK-independent mechanism. Moreover, PA was able to directly activate mTOR signaling *in vitro*. Combined, these results demonstrate that mechanical stimulation induces mTOR signaling, and protein synthesis, via an ERK-independent mechanism that potentially involves a direct interaction of PA with mTOR. Furthermore, it appears that a decrease in total 4E-BP1 may be part of the mTOR-dependent mechanism through which mechanical stimuli activate protein synthesis.

## Introduction

It is well recognized that mechanical signals play a critical role in the regulation of skeletal muscle mass and the maintenance of muscle mass contributes significantly to health and issues associated with the quality of life [1]–[3]. However, the mechanism(s) via which mechanical signals are converted into the molecular events that regulate muscle mass remain poorly defined. Nevertheless, advances in our knowledge are being made and it is becoming increasingly evident that mechanically-induced changes in muscle mass are largely driven by changes in the rate of protein synthesis [4]–[6]. Thus, identifying the molecular mechanisms that control mechanically-induced changes in protein synthesis should provide fundamental insight into how mechanical stimuli regulate muscle mass.

One mechanism that has been widely implicated in the regulation of protein synthesis involves signaling through the mammalian target of rapamycin (mTOR) [7]. For instance, mTOR-mediated phosphorylation of the eukaryotic initiation factor (eIF) 4E binding protein 1 (4E-BP1) results in the recruitment of elF4G to the 5′ end of most mRNAs, and thereby, promotes the initiation of protein synthesis [8], [9]. Furthermore, mTOR can phosphorylate, and activate, the p70 ribosomal protein S6 kinase (p70^s6k^). Active p70^s6k^ can, in-turn, promote an increase in the helicase activity of eIF4A, and thus, provide an additional stimulus for the initiation of protein synthesis [10].

mTOR is a Serine/Threonine kinase that is typically found in characteristically distinct multi-protein complexes. For example, the mTOR complex 1 (mTORC1) contains unique accessory proteins such as the regulatory-associated protein of mTOR (Raptor). On the other hand, the mTOR complex 2 (mTORC2) contains a protein called Rictor, but not Raptor. Furthermore, signaling by mTORC1, unlike mTORC2, is highly sensitive to inhibition by the drug rapamycin [11]. This is important because numerous studies have shown that rapamycin inhibits not only the mechanical activation of mTOR-dependent signaling events, but also mechanically-induced increases in the rate of protein synthesis and ultimately growth [12]–[16]. Hence, it has become widely concluded that signaling by mTOR (presumably mTORC1) is a key determinant in the mechanical regulation of protein synthesis and muscle mass; however, the mechanism(s) via which mechanical signals induce mTOR signaling remain vaguely defined.

One mechanism that has been implicated in the mechanical activation of mTOR signaling, but has not yet been directly tested, involves signaling by the extracellular regulated kinase (ERK) [17]. For example, previous studies have demonstrated that ERK can induce mTOR signaling via the phosphorylation of proteins such as tuberous sclerosis complex 2 (TSC2) and Raptor [18]–[20]. Previous studies have also shown that mechanical stimuli can induce a rapid and prolonged phosphorylation/activation of ERK, and this occurs concomitantly with the activation of mTOR signaling [13], [17]. Furthermore, Miyazaki *et al*. (2011) recently demonstrated that mechanical overload induces an increase in the phosphorylation of TSC2 on the S664 residue, which is a site that can be phosphorylated by ERK, and potentially promotes the activation of mTOR signaling. Combined, all of these studies suggest that signaling through ERK could be an important part of the mechanism via which mechanical signals activate mTOR signaling.

A potential role of ERK is also highlighted by previous studies which suggest that phosphatidic acid (PA) may be involved in the mechanical activation of mTOR signaling. Specifically, work from our lab has shown that mechanical stimuli induce an increase in the concentration of PA [21], [22]. Furthermore, several groups have shown that PA can induce the activation of mTOR signaling [21], [23], [24]; however, the mechanism(s) via which PA activates mTOR signaling have not been clearly defined. Currently, the most widely accepted mechanism involves direct binding of PA to mTOR [23], [25], [26]. Yet, an alternative mechanism has recently been proposed, in which, PA activates mTOR signaling via the induction of signaling through ERK [27]. Indeed, a number of studies support this idea. For example, PA can bind and activate Raf which, in-turn, promotes signaling through the mitogen activated protein kinase kinase (MEK)/ERK pathway [28]–[30]. Furthermore, PA can activate signaling through Raf/MEK/ERK via the recruitment of Son of Sevenless to the plasma membrane [31]. Therefore, it remains highly possible that a mechanically-induced increase in PA promotes the activation of mTOR signaling via an ERK-dependent mechanism.

Taken together, the aforementioned studies suggest that ERK could play an important role in the mechanical activation of mTOR signaling. However, limitations with the *in-vivo* administration of MEK/ERK inhibitors, such as U0126, have hampered a direct investigation into this potentially important mechanism [17]. Therefore, in this study, we attempted to overcome these limitations by employing an *ex-vivo* model of mechanical stimulation in which ERK inhibition was fully effective. Combined, our results demonstrate that the mechanical activation of mTOR signaling, and protein synthesis, occur through an ERK-independent mechanism that potentially involves a direct interaction of PA with mTOR.

## Methods

### Materials

1,2-dioctanoyl-*sn*-glycero-3-phosphate (C8 PA), egg L-*α*-phosphatidic acid (egg PA), and 1-palmitoyl-2-oleoyl-*sn*-glycero-3-phosphocholine (PC) were purchased from Avanti Lipids (Alabaster, AL, USA). For cell stimulation experiments, C8 PA and egg PA were prepared by drying lipids under nitrogen gas and dissolving in PBS with 3 min of water bath sonication at concentration of 0.6 mM and 6 mM respectively. For *in-vitro* stimulation experiments, C8 PA vesicles (50% C8 PA +50% PC) or PC vesicles (100% PC) were prepared by drying lipids as described above and dissolving in vesicle buffer (150 mM NaCl and 10 mM Tris pH 8.0) with 5 min of water bath sonication at a concentration of 6 mM. U0126 was purchased from Cell Signaling (Danvers, MA, USA) and dissolved in DMSO at concentration of 50 mM or 20 mM before adding to the *ex-vivo* organ culture media or cell culture media, respectively. Rapamycin was purchased from LC laboratories (Woburn, MA, USA) and dissolved in DMSO at a concentration of 150 µM before adding to *ex-vivo* organ culture media. Acetic acid, ethyl acetate, trimethylpentane and trichloroacetic acid were purchased from Fisher Chemical (Fair Lawn, NJ, USA). ^3^H-Myristic acid and ^3^H-Phenylalanine were purchased from Perkin Elmer (Waltham, MA, USA). Rabbit anti-phospho-p70^s6k^ (Thr389) and anti-total 4E-BP1 (R-113, used only for Figure S2) were purchased from Santa Cruz Biotechnologies (Santa Cruz,CA, USA). Peroxidase-conjugated anti-rabbit IgG (H+L) was purchased from Vector Laboratories (Burlingame, CA, USA). All other antibodies including rabbit anti-phospho-p70^s6k^ (Thr421/Ser424), anti-total p70^s6k^, anti-phospho-ERK1/2 (Thr202/Tyr204), anti-total ERK1/2, anti-phospho-S6 (Ser240/244), anti-phospho-S6 (Ser235/236), anti-total S6, anti-phopho-4E-BP1 (Thr37/46), anti-phospho-4E-BP1 (Ser65) [note: the Thr37/46 and Ser65 residues in human are equivalent to the Thr36/45 and Ser64 residues in mouse, respectively], anti-total 4E-BP1 (53H11), anti-phospho-PKB (Ser473), and anti-total PKB were purchased from Cell Signaling (Danvers, MA, USA).

### Animal Care and Use

Eight- to ten-week-old inbred or outbred (Taconic, NY, USA) FVB/N male mice were used for all experiments. For inbred mice, wild type FVB/N mice were bred with hemizygotic FVB/N mice that contain human skeletal actin promoter-driven expression of a FLAG-tagged rapamycin-resistant (Ser2035Thr) mutant of mTOR [32]. The offspring were genotyped with tail snips by PCR to identify null (wild type) and hemizygotic (RR-mTOR) mice. The mice were anaesthetized with an intraperitoneal injection of ketamine (100 mg/kg) and xylazine (10 mg/kg) before all surgical procedures. After muscle collection, mice were sacrificed by cervical dislocation under anesthesia. The collected muscles were frozen in liquid nitrogen immediately or after washing when necessary. All animals were housed in a room maintained at 25°C with a 12 h:12 h light–dark cycle and received food and water ad libitum. All methods were approved by the Institutional Animal Care and Use Committee of the University of Wisconsin-Madison under protocol # V01324.

### Organ Culture and Mechanical Stimulation

Mouse extensor digitorum longus (EDL) muscles were placed in an *ex-vivo* organ culture system which consisted of a refined myograph apparatus (Kent Scientific, Torrington, CT) and an organ culture bath as previously described [14]. In most experiments, the bath incubation media consisted of Krebs Henseleit Buffer (120 mM NaCl, 4.8 mM KCl, 25 mM NaHCO3, 2.5 mM CaCl2, 1.2 mM KH2PO4, 2 mM MgSO4, and 5 mM HEPES) supplemented with 1X MEM amino acid mixture (Invitrogen, Carlsbad, CA) and 25 mM glucose. For experiments involving the measurement of protein synthesis, high glucose DMEM was used for the incubation media (HyClone, Logan, UT, USA). In all cases, the media was maintained at 37°C with continuous 95% O_2_ and 5% CO_2_ gassing, and fresh media was added to the bath at 30 min intervals.

For mechanical stimulation, the EDL muscles were connected to the lever arms of a force transducer and micromanipulator, and then placed in the organ culture bath. The length of the EDL muscles were then adjusted until a passive tension of 13.5 mN was obtained (note: preliminary studies demonstrated that the optimal length (*Lo*) of EDL muscles was obtained at 13.5 mN). The muscles were then subjected to intermittent 15% passive stretch as a source of mechanical stimulation or held static at *Lo* as a control condition as previously described [14].

### Cell Culture

For stimulation experiments, wild type C2C12 myoblasts were purchased from ATCC (Manassas, VA) and cultured in growth media consisting of high glucose DMEM supplemented with antibiotics and antimycotics (100* µ*g/ml streptomycin, 100 U/ml penicillin and 0.25 *µ*g/ml amphotericin) and 10% fetal bovine serum (Gibco, Grand Island, NY, USA). These cells were plated on 6-well dishes and grown to confluence. Upon confluence, all cells were serum-starved in the absence of any antibiotics and antimycotics overnight before being subjected to experimental treatments. Cell cultures were maintained at 37°C in a humidified atmosphere of 95% air and 5% CO_2_.

### Western Blot Analysis

Collected muscles were homogenized with a Polytron in an ice-cold lysis buffer [40 mM Tris (pH 7.5), 1 mM EDTA, 5 mM EGTA, 0.5% Triton X-100, 25 mM *β*-glycerolphosphate, 25 mM NaF, 1 mM Na_3_VO_4_, 10 *µ*g/ml leupeptin, and 1 mM PMSF] and the whole homogenate was used for analysis. Myoblasts were lysed in the ice-cold buffer described above, centrifuged at 500 *g* for 5 min, and then the supernatant was used for analysis. Protein concentrations were determined with the DC protein assay kit (Bio-Rad, Hercules, CA, USA) and equal amounts of protein from each sample were dissolved in Laemmli buffer before being subjected to SDS-PAGE. Following the electrophoretic separation, proteins were transferred to a PVDF membrane, blocked with 5% milk in TBST (Tris-buffered saline, 1% Tween 20) for 1 h, and probed with rabbit primary antibodies overnight at 4°C. The membranes were then washed for 30 min in TBST and incubated with a peroxidase-conjugated anti-rabbit secondary antibody for 1 h at room temperature. Following 30 min of washing in TBST, the blots were developed on film using ECL [Pierce (Rockford, IL, USA) for Regular ECL and Amersham (Piscataway, NJ, USA) for ECL Plus]. Once the appropriate image was captured, the membranes were stained with Coomassie Blue to verify equal loading througout all lanes. Densitometric measurements of each blot were carried out using ImageJ (NIH).

### Analysis of Protein Synthesis

Protein synthesis rates were measured *ex-vivo* as previously described [14]. Briefly, EDL muscles were pre-incubated for 30 min and then subjected to 90 min of control or stretch conditions in DMEM. During the final 30 min, the media was switched to fresh DMEM containing 2.7 mM phenylalanine and 10 *µ*Ci/ml ^3^H-phenylalanine for 30 min. An aliquot of the media was saved for determining the specific activity of the phenylalanine (cpm/nmol of phenylalanine) before collecting the muscles. The muscles were then washed three times with ice-cold PBS (pH 7.5) and homogenized in 10% (w/v) trichloroacetic acid (TCA). The TCA homogenates were incubated on ice for 30 min and then centrifuged at 5000 *g* for 5 min. TCA-soluble material was decanted and the TCA-insoluble material was consecutively washed at least five times by repeating resuspension of the pellet in 10% TCA and centrifugation at 5000 *g*. The TCA-insoluble material was then dissolved in 0.15 M NaOH at 55°C with frequent vortex-mixing for 1 h. Aliquots of the sample were counted by liquid scintillation spectrometry and used for determination of protein concentration with the DC protein assay kit to yield the specific activity (cpm/mg of protein) of the TCA-insoluble proteins. The rate of protein synthesis was calculated by using the following equation:
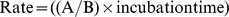
where A represents the specific activity of the TCA-insoluble material and B the specific activity of the media.

Protein synthesis rates in cells were measured by incubating the cells in DMEM media containing 1.2 mM phenylalanine and 2 *µ*Ci/ml ^3^H-phenylalanine for 30 min. After saving an aliquot of the media for determination of the phenylalanine specific activity, the reaction was terminated by rinsing the culture wells three times with ice-cold PBS and then adding 10% (w/v) TCA to the wells. The cells were incubated on ice for 30 min and collected to determine the specific activity (cpm/mg of protein) of the TCA-insoluble proteins as described above.

### Analysis of Phosphatidic Acid Concentration

The method for measuring the concentration of PA in skeletal muscles *ex-vivo* has been previously described [21]. Briefly, EDL muscles were pre-labeled in the organ culture system with media containing ^3^H-myristic acid (2.5 *µ*Ci/ml) for 2 h and then subjected to experimental treatments before being collected. The muscles were then homogenized in chloroform–methanol 2∶1 (v/v) with a polytron, and total lipids were extracted according to Folch *et al.* (1957). The extracted lipids were combined with 10 *µ*g of PA standard and aliquots were used for the measurement of radioactivity in the total lipids or spotted on LK5D silica gel plates for separation of PA by thin layer chromatography (TLC). The plates were developed with a solvent system consisting of ethyl acetate–isooctane–acetic acid–water 13∶2:3∶10 (v/v). The standard PA spots containing the ^3^H-labelled PA were visualized by iodine staining and scraped off the TLC plate to count the amount of radioactivity by liquid scintillation spectrometry. Final calculations for [PA] were made by dividing the amount of radioactivity in the PA spot by the amount of radioactivity in the total lipid extract.

### *In-vitro* mTOR Kinase Activity Assay

Mouse wild type C2C12 myoblasts were cultured as described above. C2C12 myoblasts stably expressing FLAG-tagged mTOR were obtained from Dr. Jie Chen (Department of Cell and Developmental Biology, University of Illinois, Urbana IL) and maintained in DMEM containing 0.2 mg/ml G418 (HyClone). G418 was not included in the media after the cells had been plated for the experiments. Upon confluence, cells were serum-starved overnight and subsequently collected in ice cold CHAPS lysis buffer [40 mM HEPES pH. 7.4, 2 mM EDTA, 0.3% CHAPS, 10 mM sodium pyrophosphate, 10 mM β-glycerophosphate, and 1 tablet of EDTA-free protease inhibitors (Roche) per 25 mL]. Fresh cell lysates were centrifuged at 2500 g for 5 min, and 400 µg of protein from the supernatant was diluted to a volume of 0.5 mL with fresh ice cold CHAPS lysis buffer. Samples were immunoprecipitated for the FLAG tag by incubating with 10 µL of EZview red ANTI-FLAG M2 agarose affinity gel beads (Sigma-Aldrich, St Louis, MO) and gently rocking at 4°C for 3 h. Following the incubation, the beads were pelleted by centrifugation at 500 g for 30 sec and washed 3 times with fresh ice cold Wash buffer (40 mM HEPES pH. 7.4, 150 mM NaCl, 2 mM EDTA, 0.3% CHAPS, 10 mM sodium pyrophosphate, 10 mM β-glycerophosphate, and 1 tablet of EDTA-free protease inhibitors per 25 ml). The beads were then washed 2 times with Kinase Wash buffer (25 mM HEPES pH 7.4 and 20 mM KCl).

For lipids stimulation, C8 PA or PC vesicles were prepared as described in the *Materials* section. The vesicles were diluted to 150 µM in 1.5-fold concentrated mTOR kinase assay buffer (see below), and 10 µL of this solution was incubated with the immunoprecipitates at 30°C for 15 min. Then, mTOR kinase activity towards purified GST-tagged p70 [33], [34] was initiated by adding 5 µL of 750 µM ATP to the immuoprecipitates which yielded a final mTOR kinase assay buffer that contained (25 mM HEPES pH 7.4,50 mM KCl, 10 mM MgCl2, 250 µM ATP, 50 ng GST-p70, and 100 µM C8 PA or PC vesicles). After 20 min of the reaction, the kinase assay was terminated by the addition of 25 µL of 2X Laemmli Buffer. Samples were boiled for 5 min and then subjected to western blot analysis.

### Statistical Analysis

All values are expressed as means (+ SEM in graphs). Statistical significance was determined by using ANOVA, followed by Student–Newman–Keuls *post hoc* analysis. Differences between groups were considered significant when P≤0.05. All statistical analyses were performed on SigmaStat software (San Jose, CA, USA).

## Results

### Mechanical Stimulation Activates mTOR Signaling via an ERK-independent Mechanism

**Figure 1:**
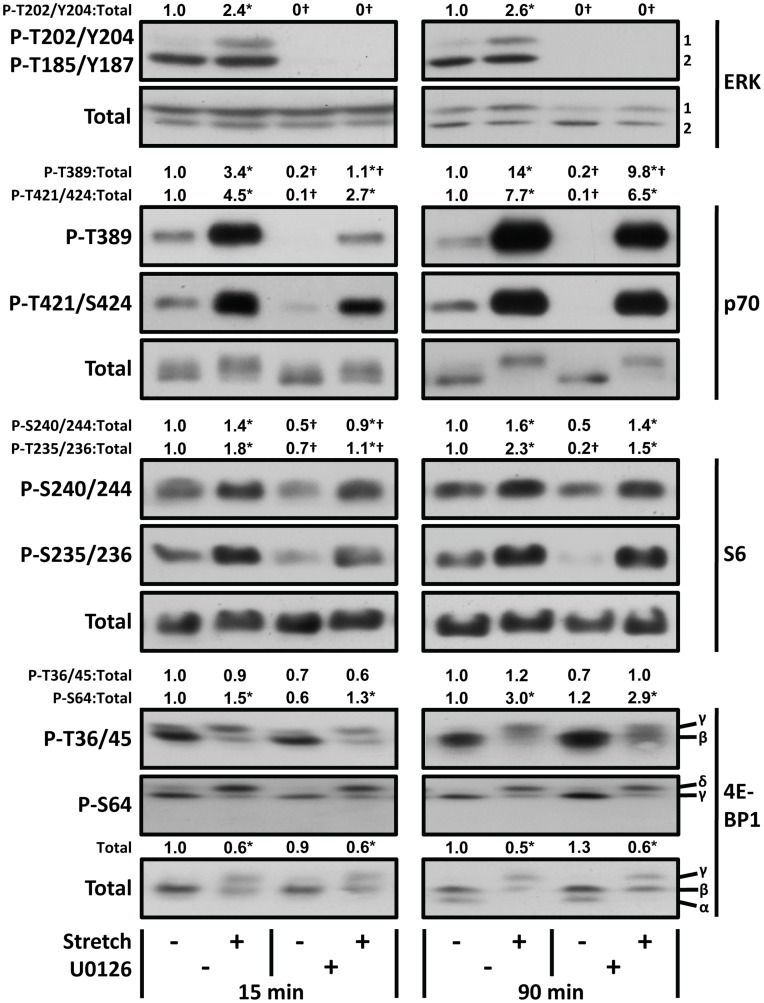
Mechanical stimulation activates mTOR signaling via an ERK-independent mechanism. EDL muscles were held at optimal length (*Lo*) in an *ex-vivo* organ culture system and pre-incubated with 50 µM U0126 (U0126+) or the vehicle (U0126 –, DMSO) for 30 min. The muscles were then subjected to 15 or 90 min of intermittent 15% stretch or held static at *Lo* as a control condition. Muscles were collected at the end of the 15 or 90 min interval and subjected to western blot analysis for phosphorylated (P) and total ERK, p70, S6, and 4E-BP1. The total amount, and phospho:total ratios, of each protein were measured and then expressed relative to the values obtained in the time-matched vehicle control samples (U0126 –, Stretch –). All values are presented as the mean (n = 3−6 per group). *Significantly different from the drug-matched control group, †Significantly different from the stimulation-matched vehicle group, P≤0.05.

As stated in the introduction, there are several lines of evidence which suggest that ERK may play a role in the mechanical activation of mTOR signaling. To test this possibility, we employed an *ex-vivo* organ culture system in which mouse EDL muscles were mechanically stimulated with intermittent passive stretch. In this model, we determined that both 15 and 90 min mechanical stimulation was sufficient to induce an increase in the phosphorylation of ERK1 (T202/Y204). Furthermore, in this system, the MEK/ERK inhibitor U0126 was fully capable of inhibiting signaling through ERK (Figure 1). Combined, these results demonstrated that our *ex-vivo* system would enable us to define the role of ERK in the mechanical activation of mTOR signaling.

Changes in the phosphorylation status of molecules such as p70^s6k^, ribosomal S6 protein (S6), and 4E-BP1, are commonly used as markers of mTOR activity. For example, the T389 residue on p70^s6k^ is a widely accepted mTOR-specific phosphorylation site [35]. On the other hand, the T421/S424 residues of p70^s6k^ contain the consensus “S/T-P” sequence which can be phosphorylated by proline-directed kinases such as ERK [36]. Therefore, we measured the phosphorylation status of the T389 and T421/S424 residues. Our results indicated that as little as 15 min of mechanical stimulation was sufficient to induce phosphorylation on these sites, and the magnitude of this effect was further amplified with 90 min of mechanical stimulation. Interestingly, when signaling through ERK was inhibited with U0126, the basal levels of T389 and T421/S424 phosphorylation were significantly reduced; however, U0126 did not block the mechanically-induced increase in the phosphorylation of these sites at either time point. Combined, these results indicate that ERK plays a role in maintaining basal levels of T389 and T421/S424 phosphorylation, but it is not necessary for a mechanically-induced increase in the phosphorylation of these sites.

As mentioned above, the phosphorylation status of S6 is also commonly used as a marker of mTOR activity, and previous studies have shown that S6 is predominantly phosphorylated by p70^s6k^ on residues such as S235/236 and S240/244 [37], [38]. Furthermore, phosphorylation of the S235/236 residues, unlike the S240/244 residues, can also be mediated by ERK-dependent activation of the p90 ribosomal S6 kinase (RSK) [39], [40]. Thus, we were interested in determining if mechanical stimulation would induce an increase in S6 phosphorylation, and if ERK would be necessary for this event. As shown in Figure 1, our results indicated that 15 min of mechanical stimulation significantly increased S6 phosphorylation on both the S235/236 and S240/244 residues, and the magnitude of this effect was similarly maintained after 90 min of mechanical stimulation. In the presence of U0126, basal levels of S235/236 and S240/244 phosphorylation were significantly reduced, but U0126 did not block the mechanically-induced increase in the phosphorylation of these residues. Hence, it can be concluded that signaling through ERK plays a role in maintaining basal levels of S235/236 and S240/244 phosphorylation, but it is not necessary for a mechanically-induced increase in the phosphorylation of these sites.

In order to further clarify the role of ERK in the mechanical activation of mTOR signaling, we measured the phosphorylation status of 4E-BP1. For example, we measured changes in gel mobility as a marker of global changes in 4E-BP1 phosphorylation [41]. As shown in Figure 1, both 15 and 90 min of mechanical stimulation induced an increase in the appearance of the slower migrating/hyper-phosphorylated γ band, and inhibition of ERK with U0126 did not alter this affect. We also measured the phosphorylation of the T36/45 residues on 4E-BP1. Importantly, numerous studies have shown that mTOR can directly phosphorylate these residues [42], [43], but much to our surprise, neither mechanical stimulation, nor U0126, altered the amount of T36/45 phosphorylation. On the other hand, 15 min of mechanical stimulation induced a significant increase in the phosphorylation of 4E-BP1 on the S64 residue, and the magnitude of this effect was further amplified with 90 min of mechanical stimulation. However, U0126 did not affect the phosphorylation of S64 in either the basal or mechanically-stimulated states. Finally, we also found that mechanical stimulation induced a significant loss in the abundance of total 4E-BP1, and again, inhibition of ERK with U0126 did not alter this effect. Note: a mechanically-induced loss of 4E-BP1 was also detected with an antibody that was raised against a different antigenic peptide (Figure S2). Taken together, these results demonstrate that mechanical stimulation induces an increase in 4E-BP1 phosphorylation on sites such as S64, as well as a loss of total 4E-BP1, and signaling through ERK is not necessary for these events.

### Validation of Mechanically-induced mTOR-dependent Signaling Events

**Figure 2:**
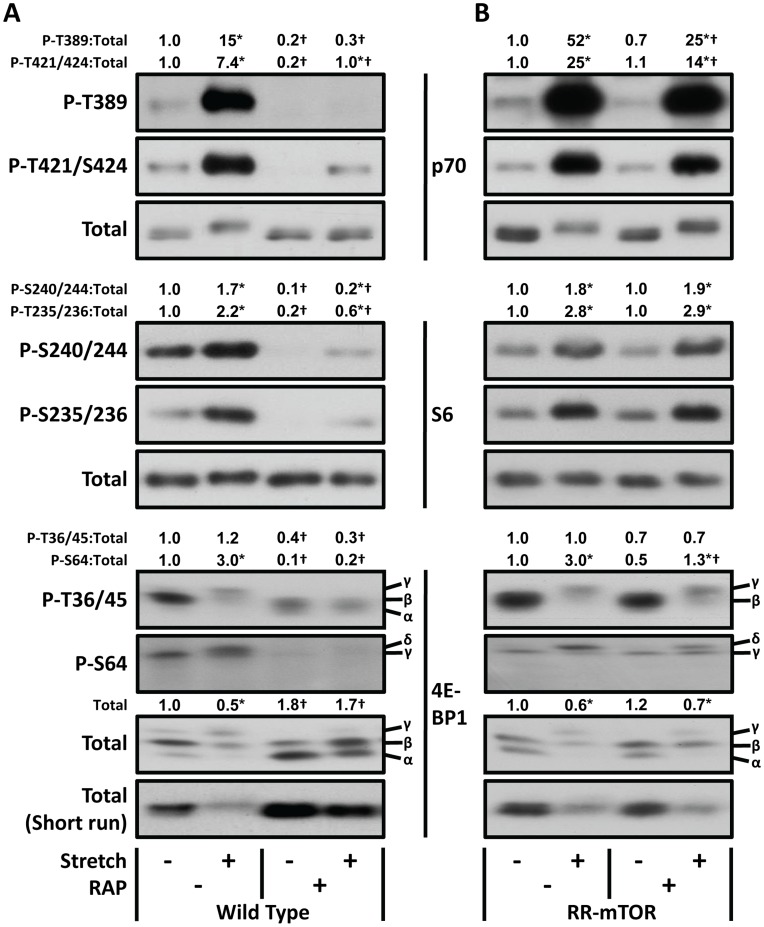
Validation of mTOR-dependent signaling events that are induced by mechanical stimulation. (**A**) EDL muscles from wild type mice, and (**B**) transgenic mice with muscle specific expression of a rapamycin-resistant mutant of mTOR (RR-mTOR), were held at *Lo* and pre-incubated with 150 nM rapamycin (RAP +) or the vehicle (RAP –, DMSO) for 30 min. The muscles were then subjected to 90 min of stretch or control conditions followed by western blot analysis for phosphorylated (P) and total p70, S6, and 4E-BP1. The total amount, and phospho:total ratios, of each protein were measured and then expressed relative to the values obtained in the genotype-matched vehicle control samples (RAP–, Stretch–). Note: total 4E-BP1 gels were also run under conditions that minimize the gel mobility shift (short-run). All values are presented as the mean (n = 3−5 per group). *Significantly different from the drug-matched control group, †Significantly different from the stimulation-matched vehicle group, P≤0.05.

As previously noted, changes in the phosphorylation status of molecules, such as p70^s6k^, S6, and 4E-BP1, are commonly used as markers of mTOR activity. However, many of the phosphorylation sites on these molecules can be regulated by both mTOR-dependent and mTOR-independent events [16], [36], [40], [44]. Hence, we set out to determine if the markers analyzed were indeed valid markers of mechanically-induced mTOR signaling. To accomplish this, we first used a supramaximal dose of rapamycin (150 nM) to inhibit signaling by mTOR in muscles from wild type mice. As shown in Figure 2A, this dose of rapamycin eliminated the mechanically-induced increase in p70^s6k^ T389 and 4E-BP1 S64 phosphorylation, suggesting that these events occur through an mTOR-dependent mechanism. Interestingly, the mechanically-induced decrease in total 4E-BP1 was also eliminated by rapamycin, which suggests that the loss of 4E-BP1 may be a previously unrecognized mTOR-dependent signaling event. On the other hand, rapamycin did not prevent mechanical stimulation from inducing an increase in the phosphorylation of the p70^s6k^ T421/S424, S6 S235/236 and S6 S240/244 residues. Thus, it can be concluded that rapamycin-insensitive/mTOR-independent mechanism(s) are at least partially responsible for the mechanically-induced phosphorylation of these residues.

Like all pharmacological inhibitors, rapamycin can potentially exert non-specific (mTOR-independent) actions. For example, it has been shown that rapamycin can bind and sequester the FKBP12 protein and this can interfere with the role that FKBP12 plays in the function of the ryanodine receptor and signaling by members of the transforming growth factor-β superfamily [45]–[47]. Thus, we wanted to determine if mTOR was indeed responsible for the inhibitory effects of rapamycin. To accomplish this, we employed transgenic mice that express endogenous wild type mTOR in conjunction with overexpression of a rapamycin-resistant mutant of mTOR (RR-mTOR). Specifically, the RR-mTOR transgene contains a S2035T mutation located within the FKBP12-rapamycin complex binding domain of mTOR. The S2035T mutation prevents the interaction of mTOR with the FKBP12-rapamycin complex, and thereby, confers resistance to the inhibitory effects that rapamycin normally exerts on mTOR signaling [48], [49]. As shown in Figure 2B, when muscles from RR-mTOR mice were subjected to mechanical stimulation in the presence of rapamycin, the mechanically-induced increase in p70^s6k^ T389 and 4E-BP1 S64 phosphorylation, and the decrease in total 4E-BP1, were all effectively rescued from the inhibitory actions of rapamycin. Therefore, it can be concluded that these events are indeed valid markers of mechanically-induced signaling by mTOR.

### Mechanical Stimulation Induces Protein Synthesis via an ERK-independent Mechanism

**Figure 3:**
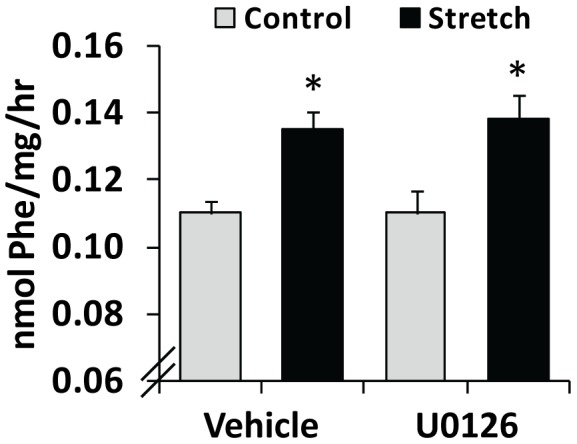
Mechanical stimulation induces an increase in protein synthesis via an ERK-independent mechanism. EDL muscles were held at *Lo* and pre-incubated with 50 µM U0126 or the vehicle (DMSO) for 30 min. The muscles were then subjected to 90 min of stretch or control conditions, and protein synthesis rates were measured during the final 30 min. All values are presented as the mean + SEM (n = 4−7 per group). *Significantly different from the drug-matched control group, P≤0.05.

Previous studies have indicated that signaling through mTOR is necessary for a mechanically-induced increase in the rate of protein synthesis, and that the activation of mTOR signaling is sufficient to induce protein synthesis [13]–[15], [50]. In addition, the results presented above indicate that ERK is not necessary for the mechanical activation of mTOR signaling. Combined, these points suggest that mechanical stimulation should induce an increase in protein synthesis via an ERK-independent mechanism. However, signaling by ERK can also control protein synthesis by regulating mTOR-independent molecules such as RSK and the mitogen-activated protein kinase-interacting S/T kinase [51], [52]. Thus, we wanted to determine if signaling through ERK is necessary for a mechanically-induced increase in the rate of protein synthesis. To test this, we subjected muscles to 90 min of mechanical stimulation and then measured rates of protein synthesis during the final 30 min of stimulation. Interestingly, we could not observe a mechanically-induced increase in the rate of protein synthesis when muscles were incubated with the same media [Krebs Henseleit Buffer (KHB) supplemented with amino acids and glucose, see methods] that was used to obtain the signaling data presented in Figures 1 and 2 (Figure S1). However, we have previously demonstrated that mechanical stimulation in our organ culture system induces protein synthesis when muscles are incubated with DMEM media [14]. Therefore, we performed our protein synthesis measurements on muscles that were incubated with DMEM. Before doing this, we confirmed that mechanical stimulation induced an ERK-independent activation of mTOR signaling when muscles were incubated with DMEM (Figure S2). We then performed our protein synthesis measurements and, consistent with our previous studies, we found that mechanical stimulation induced an increase in the rate of protein synthesis. However, inhibition of ERK with U0126 did not prevent the mechanically-induced increase in protein synthesis (Figure 3). Thus, it can be concluded that signaling through ERK is not necessary for a mechanically-induced increase in protein synthesis.

### PA Induces mTOR Signaling via an ERK-independent Mechanism

**Figure 4:**
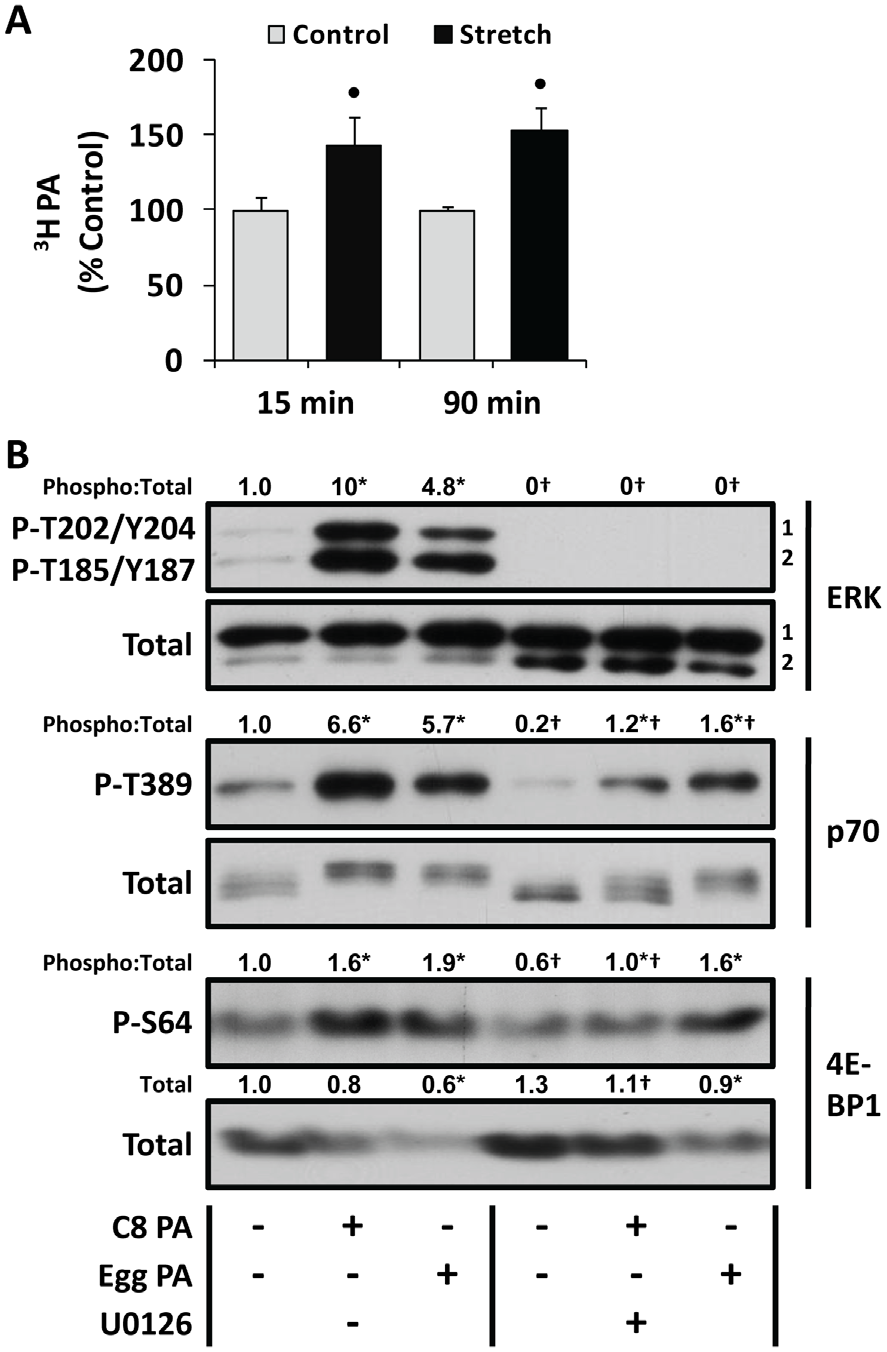
Exogenous phosphatidic acid activates mTOR signaling via an ERK-independent mechanism. (**A**) EDL muscles were held at *Lo* and pre-labeled with [^3^H]-myristic acid for 2 h. The muscles were then subjected to 15 or 90 min of stretch or control conditions. Muscles were collected at the end of the 15 or 90 min interval, and the amount of ^3^H-labeled PA (^3^H PA) was measured and then expressed as a percentage of time-matched control values. The values are presented as the mean + SEM (n = 4−7 per group). • Significantly different from the time-matched control group. (**B**) C2C12 myoblasts were serum-starved overnight and then pre-incubated with 50 µM U0126 (U0126+) or the vehicle (U0126 –, DMSO) for 30 min, followed by 20 min stimulation with 30 µM C8 PA, 300 µM Egg PA or the vehicle (Control, PBS). The samples were then subjected to western blot analysis for phosphorylated (P) and total ERK, p70, and 4E-BP1. The total amount, and phospho:total ratios, of each protein were measured and then expressed relative to the values obtained in the vehicle control samples (U0126 –, PBS). All values are presented as the mean and were obtained from three independent experiments (n = 3−5 per group). *Significantly different from the drug-matched control group, †Significantly different from the stimulation-matched vehicle group, P≤0.05.

Previous studies have suggested that a mechanically-induced increase in PA is necessary for the activation of mTOR signaling [21], [22]. Furthermore, an increase in PA is capable of inducing the activation of ERK, which could then induce signaling by mTOR [18], [29], [31]. Based on these points, we initially envisioned the potential for a mechanism in which a mechanically-induced increase in PA would promote signaling through ERK and this, in-turn, would induce the activation of mTOR signaling. However, our results demonstrated that the mechanical activation of mTOR signaling occurred through an ERK-independent mechanism. Thus, we reasoned that if PA is actually involved in the mechanical activation of mTOR signaling, then PA should also promote the activation of mTOR signaling via an ERK-independent mechanism. To test this, we first wanted to confirm our previous observation that mechanical stimulation induces an increase in PA. As shown in Figure 4A, both 15 and 90 min of mechanical stimulation resulted in a significant increase in ^3^H-myristic acid labeled PA.

Next, we wanted to determine if PA activates mTOR signaling via an ERK-independent mechanism. Importantly, we have previously confirmed that exogenous PA can induce mTOR signaling in C2C12 myoblasts [21]. Thus, to accomplish our goal, we stimulated C2C12 myoblasts with different species of exogenous PA (C8 PA and Egg PA) in the presence or absence of U0126. It should be noted that at least some of exogenous PA can be hydrolyzed into lysophosphatidic acid (LPA) by the phospholipase A present in the cell culture media, and this could promote additional LPA receptor-mediated activation of ERK [27]. Consistent with this possibility, we found that treatment with both species of exogenous PA induced a much more robust increase in ERK phosphorylation than what was observed with mechanical stimulation (Figure 4B). Furthermore, the robust activation of ERK by C8 PA was found to be necessary for C8 PA to induce an increase in protein synthesis (Figure S3). However, inhibition of ERK with U0126 did not prevent the ability of PA to induce the markers of mechanically-induced mTOR signaling, such as p70^s6k^ T389 and 4E-BP1 S64 phosphorylation, or the loss of total 4EBP1 (Figure 4B). These results indicate that, in C2C12 myoblasts, exogenous PA induces mTOR-dependent signaling events via an ERK-independent mechanism.

### PA can Directly Activate mTOR Signaling *in-vitro*

**Figure 5:**
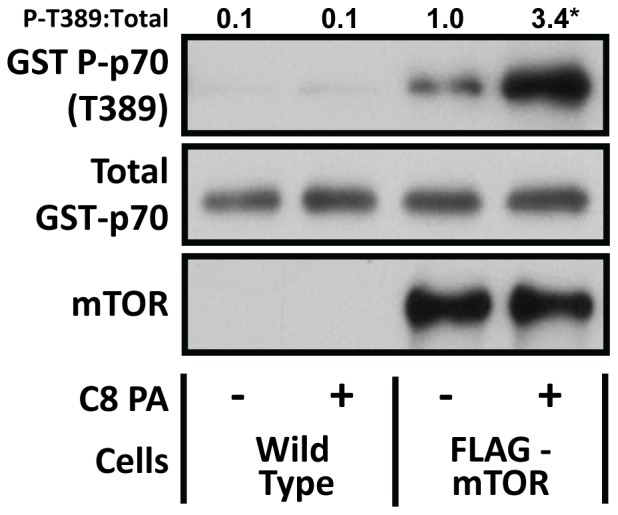
Phosphatidic acid directly promotes mTOR kinase activity *in-vitro*. Wild type C2C12 myoblasts and C2C12 myoblasts stably expressing FLAG-tagged mTOR (FLAG-mTOR) were serum starved overnight, collected, and then the cell lysates were subjected to immunoprecipitation against the FLAG epitope. The immunoprecipitates were incubated for 15 min with either 150 µM C8 PA vesicles (50% C8 PA +50% PC) or 150 µM phosphatidylcholine (PC) vesicles (100% PC) as a control condition. mTOR kinase activity was then assayed with GST-p70 as a substrate. The resulting samples were subjected to western blot analysis, and the phospho:total ratios for GST-p70 were expressed relative to the values obtained in the PC treated FLAG-mTOR group. All values are presented as the mean and were obtained from at least three independent experiments (n = 5−8 per group). *Significantly different from the cell type-matched control group, P≤0.05.

Our observation that PA induces mTOR signaling via an ERK-independent mechanism prompted us to explore the possibility that PA might directly activate mTOR signaling [23], [25], [26]. To test this, we employed wild type C2C12 myoblasts and C2C12 myoblasts that stably express FLAG-tagged wild type mTOR [53]. Lysates from these cells were first subjected to immunoprecipitation against the FLAG tag, and as shown in Figure 5, only the immunoprecipitates from the FLAG-mTOR myoblasts contained mTOR. Next, the immunoprecipitates were incubated with lipid vesicles composed of 50% C8 PA and 50% PC, or 100% PC as a control condition. The immunoprecipitates were then subjected to an *in-vitro* mTOR kinase activity assay in which p70^s6k^ T389 phosphorylation was used as a readout of mTOR activity [33]. The results demonstrated that PA can induce an increase in p70^s6k^ T389 phosphorylation, and this only occurred in the immunoprecipitates obtained from the FLAG-mTOR cells. This is an important point because it established that the PA-induced increase in p70^s6k^ T389 phosphorylation required the presence of mTOR and, therefore, did not result from the presence of a non-specific kinase that was pulled down during the immunoprecipitation procedure. Thus, it can be concluded that PA is capable of directly activating mTOR signaling *in-vitro*.

## Discussion

In this study we employed an *ex-vivo* passive stretch model of mechanical stimulation to determine if signaling through ERK is necessary for the mechanical activation of mTOR signaling and protein synthesis. Based on the results, it can be concluded that ERK’s contribution to these events, if any, is very limited. However, we also found that the basal level of mTOR signaling was severely impaired by ERK inhibition, which suggests that ERK is indeed a potent regulator of mTOR signaling in skeletal muscle. Hence, the seemingly negligible role of ERK in the mechanical activation of mTOR signaling is likely due, at least in part, to the relatively small increase in signaling through ERK that was induced by our model of mechanical stimulation (≈2-fold). However, a more robust increase in signaling through ERK has been observed with other types of mechanical stimulation. For example, Martineau and Gardiner 2001, demonstrated that concentric, isometric, and eccentric contractions produce a 3-, 4-, and 5-fold increase in signaling through ERK, respectively [54]. Therefore, we cannot fully exclude the possibility that ERK may contribute to the activation of mTOR signaling, and protein synthesis, in other models of mechanical stimulation as previously suggested by Miyazaki *et al.,* 2011 [17]. Nevertheless, our results do clearly reveal that mechanical stimuli can induce mTOR signaling, and protein synthesis, through an ERK-independent mechanism.

To date, numerous studies have attempted to identify the molecular mechanisms that are involved in the mechanical activation of mTOR signaling [55], [56]. Based on these studies, it has been concluded that mechanical stimuli activate mTOR signaling through a unique mechanism that does not require typical candidates such as phosphoinositide 3-kinase, protein kinase B, exogenous nutrients, protein kinase C, phosphoinositide-specific phospholipase C, or changes in intracellular calcium [14], [21], [22], [57]. On the other hand, several lines of evidence suggest that PA may be involved [21], [22]. For example, the results of this study demonstrate that; i) mechanical stimuli can induce an increase in PA, ii) stimulating cells with PA is sufficient to induce mTOR signaling, and iii) PA can directly activate mTOR signaling *in-vitro*. Furthermore, like mechanical stimuli, we found that PA induces mTOR signaling through an ERK-independent mechanism. All of these observations are consistent with a model in which mechanical stimuli induce an increase in PA, and the newly formed PA then binds and activates mTOR. Although intriguing, additional studies will be needed to test the validity of this concept.

In this study, we also used RR-mTOR mice to identify valid markers of mechanically-induced mTOR signaling. For example, the RR-mTOR mice enabled us to demonstrate that a mechanically-induced increase in p70^s6k^ T389 phosphorylation is fully dependent on mTOR. Conversely, we found that other commonly used markers of mTOR signaling, such as S6 S235/236 and S6 S240/244 phosphorylation, were at least partially induced via a rapamycin-insensitive/mTOR-independent mechanism. Consistent with these results, another recent study also demonstrated that rapamycin does not block mechanical overload-induced increases in S6 S235/236 and S6 S240/244 phosphorylation [17]. This is particularly interesting because a previous study with p70^s6k1^ and p70^s6k2^ double knockout mice demonstrated that S6 S240/244 phosphorylation requires p70^s6k^ activity [39]. Thus, our results might indicate that mechanical stimulation induces a partially rapamycin-resistant activation of p70^s6k^, the activation of unrecognized S6 kinase(s), or an inhibition of S6 phosphatases. Furthermore, our observations illustrate that caution should be used when interpreting changes in S6 S235/236 or S6 S240/244 phosphorylation as markers of mTOR signaling.

The RR-mTOR mice also enabled us to demonstrate that mTOR is necessary for mechanical stimulation to induce an increase in 4E-BP1 S64 phosphorylation, and a decrease in total 4E-BP1. However, much to our surprise, mechanical stimulation did not alter 4E-BP1 T36/45 phosphorylation. This observation was surprising because numerous studies have shown that, when activated, the rapamycin-sensitive mTORC1 can directly phosphorylate both p70^s6k^ T389 and 4E-BP1 T36/45 residues [58], [59]. Furthermore, an extensively large number of studies have shown that the classical agonists of mTORC1 signaling (e.g. growth factors and nutrients) induce an increase in both p70^s6k^ T389 and 4E-BP1 T36/45 phosphorylation [33], [60], [61]. Hence, observing a robust increase in p70^s6k^ T389 phosphorylation, in conjunction with no change in 4E-BP1 T36/45 phosphorylation, was highly unexpected. Although the reason for this disparity is not known, a recent study by Yip et al. 2010 may have revealed some important clues. Specifically, Yip et al. demonstrated that rapamycin inhibits the ability of immunopurified mTOR to phosphorylate p70^s6k^ T389 and 4E-BP1 T36/45 [58]. Furthermore, the presence of raptor was found to be necessary for mTOR to phosphorylate 4E-BP1 T36/45. However, contrary to previous assumptions, the presence of raptor was not necessary for mTOR to phosphorylate p70^s6k^ T389. Yet, in the absence of raptor, the ability of mTOR to phosphorylate p70^s6k^ T389 was still sensitive to inhibition by rapamycin. In other words, it appears that there is a rapamycin-sensitive pool of mTOR that does not involve the classical raptor-associated mTORC1 complex. Similar to the results we observed with mechanical stimulation, this uncharacterized rapamycin-sensitive pool of mTOR can phosphorylate p70^s6k^ T389, but not 4E-BP1 T36/45. Thus, it is very tempting to speculate that mechanical stimuli activate a unique rapamycin-sensitive pool of mTOR that is distinct from mTORC1.

As mentioned in the introduction, signaling through mTOR has been widely implicated in the regulation of protein synthesis. For example, the mTOR kinase inhibitor (Torin 1) induces a greater than 60% reduction in the rate of protein synthesis when added to wild-type mouse embryonic fibroblasts (MEF). However, Torin 1 has essentially no effect on the rate of protein synthesis when added to MEFs that are deficient of 4E-BP1 and 4E-BP2 [62]. Based on these observations, it has been concluded that the 4E-BPs play a key role in the mechanism through which mTOR controls protein synthesis.

In general, mTOR is thought to control the function of the 4E-BPs by inducing changes in the phosphorylation state of the protein. For example, mTOR can directly phosphorylate the 36/45 residues of 4E-BP1, and phosphorylation on these residues allows 4E-BP1 to become further phosphorylated on residues such as S64 [43], [63]. When hyperphosphorylated, 4E-BP1 dissociates from eIF4E and this, in-turn, promotes the formation of the eIF4F complex and ultimately the initiation of protein synthesis [8], [9]. In addition to controlling 4E-BP1 phosphorylation, there is also emerging evidence which suggests that mTOR can regulate the abundance of 4E-BP1. For example, inducible knockout of mTOR in the adult myocardium results in an increase in total 4E-BP1 [64]. Furthermore, HSV-1 infection has been shown to induce a decrease in total 4E-BP1, and this effect can be prevented by rapamycin [65]. Presumably, a decrease in total 4E-BP1, and hyperphosphorylation of 4E-BP1, would result in functionally equivalent effects on protein synthesis (i.e. enhanced eIF4F complex formation). Thus, we were intrigued by our results which demonstrated that mechanical stimulation induces an mTOR-dependent decrease in total 4E-BP1. Specifically, previous studies have indicated that signaling through mTOR is necessary for a mechanically-induced increase in protein synthesis but the mechanisms through which mTOR exerts this effect have not been defined. Based on our results, it would appear that a mechanically-induced decrease in total 4E-BP1 could be part of this mechanism. Furthermore, we have measured total 4E-BP1 levels in muscles that were subjected to *in-vivo* eccentric contractions as described in [21], and again, we observed a decrease in total 4E-BP1 levels (data not shown). Based on these observations, it would appear that a mechanically-induced decrease in the total 4E-BP1 levels is a conserved event. Hence, in the future, it will be important to determine if the loss of total 4E-BP1 plays a significant role in the mechanism through which mechanical stimuli induce an increase in protein synthesis.

In summary, the results from this study demonstrate that mechanical stimulation induces mTOR signaling, and protein synthesis, via an ERK-independent mechanism that potentially involves a direct interaction of PA with mTOR. Since signaling through mTOR is necessary for a mechanically-induced increase in protein synthesis, and ultimately growth, these findings should help advance our understanding of how mechanical signals are converted into the molecular events that regulate skeletal muscle mass.

## Supporting Information

Figure S1 **Mechanical stimulation does not increase protein synthesis in muscles incubated with KHB media.** EDL muscles were held at *Lo* and pre-incubated for 30 min with KHB media containing 0.1% DMSO, 1X MEM amino acids and 25 mM glucose. The muscles were then subjected to 90 min of stretch or control conditions, and protein synthesis rates were measured during the final 30 min. All values are presented as the mean + SEM (n = 4−6 per group).
(TIF)

Figure S2 **Mechanical stimulation activates mTOR signaling via an ERK-independent mechanism in DMEM media.** EDL muscles were held at *Lo* and pre-incubated with DMEM media containing 50 µM U0126 (U0126+) or the vehicle (U0126 –, DMSO) for 30 min. The muscles were then subjected to 90 min of stretch or control conditions. Muscles were collected at the end of the 90 min interval and subjected to western blot analysis for phosphorylated (P) and total ERK, p70 and total 4EBP1. The total amount, and phospho:total ratios, of each protein were measured and then expressed relative to the values obtained in the vehicle control samples (U0126 –, Stretch –). Note: total 4E-BP1 was measured with two different antibodies, i) a monoclonal antibody from Cell Signaling that was raised against a peptide surrounding the Ser112 residues (C.S.), and ii) a polyclonal antibody from Santa Cruz that was raised against full length 4E-BP1 as the antigenic peptide (S.C.). All values are presented as the mean (n = 3−5 per group). *Significantly different from the drug-matched control group, †Significantly different from the stimulation-matched vehicle group, P≤0.05.
(TIF)

Figure S3 **Exogenous phosphatidic acid induces protein synthesis via an ERK-dependent mechanism.** C2C12 myoblasts were serum-starved overnight and then pre-incubated with 50 µM U0126 or the vehicle (DMSO) for 30 min, followed by 60 min stimulation with 30 µM C8 PA or the vehicle (PBS). Protein synthesis rates were measured during the final 30 min and expressed as a percentage of the vehicle control values. All values are presented as the mean + SEM and were obtained from five independent experiments (n = 9−11 per group). *Significantly different from the drug-matched control group. †Significantly different from the stimulation-matched vehicle group, P≤0.05.
(TIF)

## References

[1] Izumiya Y, Hopkins T, Morris C, Sato K, Zeng L, et al (2008) Fast/Glycolytic muscle fiber growth reduces fat mass and improves metabolic parameters in obese mice. Cell Metab 7: 159–172. 18249175 10.1016/j.cmet.2007.11.003 PMC2828690

[2] Proctor DN, Balagopal P, Nair KS (1998) Age-related sarcopenia in humans is associated with reduced synthetic rates of specific muscle proteins. J Nutr 128: 351S–355S. 9478023 10.1093/jn/128.2.351S

[3] Pahor M, Kritchevsky S (1998) Research hypotheses on muscle wasting, aging, loss of function and disability. J Nutr Health Aging 2: 97–100. 10993575

[4] Fitts RH, Riley DR, Widrick JJ (2000) Physiology of a microgravity environment invited review: microgravity and skeletal muscle. J Appl Physiol 89: 823–839. 10926670 10.1152/jappl.2000.89.2.823

[5] Goldberg AL, Etlinger JD, Goldspink DF, Jablecki C (1975) Mechanism of work-induced hypertrophy of skeletal muscle. Med Sci Sports 7: 185–198. 128681

[6] Vandenburgh HH, Hatfaludy S, Sohar I, Shansky J (1990) Stretch-induced prostaglandins and protein turnover in cultured skeletal muscle. Am J Physiol 259: C232–240. 2382700 10.1152/ajpcell.1990.259.2.C232

[7] Ma XM, Blenis J (2009) Molecular mechanisms of mTOR-mediated translational control. Nat Rev Mol Cell Biol 10: 307–318. 19339977 10.1038/nrm2672

[8] Hara K, Yonezawa K, Kozlowski MT, Sugimoto T, Andrabi K, et al (1997) Regulation of eIF-4E BP1 phosphorylation by mTOR. J Biol Chem 272: 26457–26463. 9334222 10.1074/jbc.272.42.26457

[9] Haghighat A, Mader S, Pause A, Sonenberg N (1995) Repression of cap-dependent translation by 4E-binding protein 1: competition with p220 for binding to eukaryotic initiation factor-4E. Embo J 14: 5701–5709. 8521827 10.1002/j.1460-2075.1995.tb00257.x PMC394685

[10] Raught B, Peiretti F, Gingras AC, Livingstone M, Shahbazian D, et al (2004) Phosphorylation of eucaryotic translation initiation factor 4B Ser422 is modulated by S6 kinases. EMBO J 23: 1761–1769. 15071500 10.1038/sj.emboj.7600193 PMC394247

[11] Zoncu R, Efeyan A, Sabatini DM (2011) mTOR: from growth signal integration to cancer, diabetes and ageing. Nat Rev Mol Cell Biol 12: 21–35. 21157483 10.1038/nrm3025 PMC3390257

[12] Bodine SC, Stitt TN, Gonzalez M, Kline WO, Stover GL, et al (2001) Akt/mTOR pathway is a crucial regulator of skeletal muscle hypertrophy and can prevent muscle atrophy in vivo. Nat Cell Biol 3: 1014–1019. 11715023 10.1038/ncb1101-1014

[13] Drummond MJ, Fry CS, Glynn EL, Dreyer HC, Dhanani S, et al (2009) Rapamycin administration in humans blocks the contraction-induced increase in skeletal muscle protein synthesis. J Physiol 587: 1535–1546. 19188252 10.1113/jphysiol.2008.163816 PMC2678224

[14] Hornberger TA, Stuppard R, Conley KE, Fedele MJ, Fiorotto ML, et al (2004) Mechanical stimuli regulate rapamycin-sensitive signalling by a phosphoinositide 3-kinase-, protein kinase B- and growth factor-independent mechanism. Biochem J 380: 795–804. 15030312 10.1042/BJ20040274 PMC1224227

[15] Kubica N, Bolster DR, Farrell PA, Kimball SR, Jefferson LS (2005) Resistance exercise increases muscle protein synthesis and translation of eukaryotic initiation factor 2Bepsilon mRNA in a mammalian target of rapamycin-dependent manner. J Biol Chem 280: 7570–7580. 15591312 10.1074/jbc.M413732200

[16] Goodman CA, Frey JW, Mabrey DM, Jacobs BL, Lincoln HC, et al (2011) The role of skeletal muscle mTOR in the regulation of mechanical load-induced growth. J Physiol 589: 5485–5501. 21946849 10.1113/jphysiol.2011.218255 PMC3240886

[17] Miyazaki M, McCarthy JJ, Fedele MJ, Esser KA (2011) Early activation of mTORC1 signalling in response to mechanical overload is independent of phosphoinositide 3-kinase/Akt signalling. J Physiol 589: 1831–1846. 21300751 10.1113/jphysiol.2011.205658 PMC3099033

[18] Ma L, Chen Z, Erdjument-Bromage H, Tempst P, Pandolfi PP (2005) Phosphorylation and functional inactivation of TSC2 by Erk implications for tuberous sclerosis and cancer pathogenesis. Cell 121: 179–193. 15851026 10.1016/j.cell.2005.02.031

[19] Roux PP, Ballif BA, Anjum R, Gygi SP, Blenis J (2004) Tumor-promoting phorbol esters and activated Ras inactivate the tuberous sclerosis tumor suppressor complex via p90 ribosomal S6 kinase. Proc Natl Acad Sci U S A 101: 13489–13494. 15342917 10.1073/pnas.0405659101 PMC518784

[20] Carriere A, Romeo Y, Acosta-Jaquez HA, Moreau J, Bonneil E, et al (2011) ERK1/2 phosphorylate Raptor to promote Ras-dependent activation of mTOR complex 1 (mTORC1). J Biol Chem 286: 567–577. 21071439 10.1074/jbc.M110.159046 PMC3013016

[21] O’Neil TK, Duffy LR, Frey JW, Hornberger TA (2009) The role of phosphoinositide 3-kinase and phosphatidic acid in the regulation of mammalian target of rapamycin following eccentric contractions. J Physiol 587: 3691–3701. 19470781 10.1113/jphysiol.2009.173609 PMC2742291

[22] Hornberger TA, Chu WK, Mak YW, Hsiung JW, Huang SA, et al (2006) The role of phospholipase D and phosphatidic acid in the mechanical activation of mTOR signaling in skeletal muscle. Proc Natl Acad Sci U S A 103: 4741–4746. 16537399 10.1073/pnas.0600678103 PMC1450240

[23] Yoon MS, Sun Y, Arauz E, Jiang Y, Chen J (2011) Phosphatidic acid activates mammalian target of rapamycin complex 1 (mTORC1) kinase by displacing FK506 binding protein 38 (FKBP38) and exerting an allosteric effect. J Biol Chem 286: 29568–29574. 21737445 10.1074/jbc.M111.262816 PMC3190997

[24] Tee AR, Anjum R, Blenis J (2003) Inactivation of the tuberous sclerosis complex-1 and -2 gene products occurs by phosphoinositide 3-kinase/Akt-dependent and -independent phosphorylation of tuberin. J Biol Chem 278: 37288–37296. 12867426 10.1074/jbc.M303257200

[25] Fang Y, Vilella-Bach M, Bachmann R, Flanigan A, Chen J (2001) Phosphatidic acid-mediated mitogenic activation of mTOR signaling. Science 294: 1942–1945. 11729323 10.1126/science.1066015

[26] Veverka V, Crabbe T, Bird I, Lennie G, Muskett FW, et al (2008) Structural characterization of the interaction of mTOR with phosphatidic acid and a novel class of inhibitor: compelling evidence for a central role of the FRB domain in small molecule-mediated regulation of mTOR. Oncogene 27: 585–595. 17684489 10.1038/sj.onc.1210693

[27] Winter JN, Fox TE, Kester M, Jefferson LS, Kimball SR (2010) Phosphatidic acid mediates activation of mTORC1 through the ERK signaling pathway. Am J Physiol Cell Physiol 299: C335–344. 20427710 10.1152/ajpcell.00039.2010 PMC2928642

[28] Ghosh S, Strum JC, Sciorra VA, Daniel L, Bell RM (1996) Raf-1 kinase possesses distinct binding domains for phosphatidylserine and phosphatidic acid. Phosphatidic acid regulates the translocation of Raf-1 in 12-O-tetradecanoylphorbol-13-acetate-stimulated Madin-Darby canine kidney cells. J Biol Chem 271: 8472–8480. 8626548 10.1074/jbc.271.14.8472

[29] Rizzo MA, Shome K, Vasudevan C, Stolz DB, Sung TC, et al (1999) Phospholipase D and its product, phosphatidic acid, mediate agonist-dependent raf-1 translocation to the plasma membrane and the activation of the mitogen-activated protein kinase pathway. J Biol Chem 274: 1131–1139. 9873061 10.1074/jbc.274.2.1131

[30] Rizzo MA, Shome K, Watkins SC, Romero G (2000) The recruitment of Raf-1 to membranes is mediated by direct interaction with phosphatidic acid and is independent of association with Ras. J Biol Chem 275: 23911–23918. 10801816 10.1074/jbc.M001553200

[31] Zhao C, Du G, Skowronek K, Frohman MA, Bar-Sagi D (2007) Phospholipase D2-generated phosphatidic acid couples EGFR stimulation to Ras activation by Sos. Nat Cell Biol 9: 706–712. 17486115 10.1038/ncb1594

[32] Ge Y, Wu AL, Warnes C, Liu J, Zhang C, et al (2009) mTOR regulates skeletal muscle regeneration in vivo through kinase-dependent and kinase-independent mechanisms. Am J Physiol Cell Physiol 297: C1434–1444. 19794149 10.1152/ajpcell.00248.2009 PMC2793064

[33] Ikenoue T, Hong S, Inoki K (2009) Monitoring mammalian target of rapamycin (mTOR) activity. Methods Enzymol 452: 165–180. 19200882 10.1016/S0076-6879(08)03611-2

[34] Goodman CA, Miu MH, Frey JW, Mabrey DM, Lincoln HC, et al (2010) A phosphatidylinositol 3-kinase/protein kinase B-independent activation of mammalian target of rapamycin signaling is sufficient to induce skeletal muscle hypertrophy. Mol Biol Cell 21: 3258–3268. 20668162 10.1091/mbc.E10-05-0454 PMC2938390

[35] Magnuson B, Ekim B, Fingar DC (2012) Regulation and function of ribosomal protein S6 kinase (S6K) within mTOR signalling networks. Biochem J 441: 1–21. 22168436 10.1042/BJ20110892

[36] Mukhopadhyay NK, Price DJ, Kyriakis JM, Pelech S, Sanghera J, et al (1992) An array of insulin-activated, proline-directed serine/threonine protein kinases phosphorylate the p70 S6 kinase. J Biol Chem 267: 3325–3335. 1737788

[37] Ferrari S, Bandi HR, Hofsteenge J, Bussian BM, Thomas G (1991) Mitogen-activated 70K S6 kinase. Identification of in vitro 40 S ribosomal S6 phosphorylation sites. J Biol Chem 266: 22770–22775. 1939282

[38] Jeno P, Ballou LM, Novak-Hofer I, Thomas G (1988) Identification and characterization of a mitogen-activated S6 kinase. Proc Natl Acad Sci U S A 85: 406–410. 3257566 10.1073/pnas.85.2.406 PMC279557

[39] Pende M, Um SH, Mieulet V, Sticker M, Goss VL, et al (2004) S6K1(−/−)/S6K2(−/−) mice exhibit perinatal lethality and rapamycin-sensitive 5′-terminal oligopyrimidine mRNA translation and reveal a mitogen-activated protein kinase-dependent S6 kinase pathway. Mol Cell Biol 24: 3112–3124. 15060135 10.1128/MCB.24.8.3112-3124.2004 PMC381608

[40] Roux PP, Shahbazian D, Vu H, Holz MK, Cohen MS, et al (2007) RAS/ERK signaling promotes site-specific ribosomal protein S6 phosphorylation via RSK and stimulates cap-dependent translation. J Biol Chem 282: 14056–14064. 17360704 10.1074/jbc.M700906200 PMC3618456

[41] Gingras AC, Kennedy SG, O’Leary MA, Sonenberg N, Hay N (1998) 4E-BP1, a repressor of mRNA translation, is phosphorylated and inactivated by the Akt(PKB) signaling pathway. Genes Dev 12: 502–513. 9472019 10.1101/gad.12.4.502 PMC316523

[42] Gingras AC, Gygi SP, Raught B, Polakiewicz RD, Abraham RT, et al (1999) Regulation of 4E-BP1 phosphorylation: a novel two-step mechanism. Genes Dev 13: 1422–1437. 10364159 10.1101/gad.13.11.1422 PMC316780

[43] Mothe-Satney I, Brunn GJ, McMahon LP, Capaldo CT, Abraham RT, et al (2000) Mammalian target of rapamycin-dependent phosphorylation of PHAS-I in four (S/T)P sites detected by phospho-specific antibodies. J Biol Chem 275: 33836–33843. 10942774 10.1074/jbc.M006005200

[44] Karim MM, Hughes JM, Warwicker J, Scheper GC, Proud CG, et al (2001) A quantitative molecular model for modulation of mammalian translation by the eIF4E-binding protein 1. J Biol Chem 276: 20750–20757. 11278829 10.1074/jbc.M011068200

[45] Osman B, Doller A, Akool el S, Holdener M, Hintermann E, et al (2009) Rapamycin induces the TGFbeta1/Smad signaling cascade in renal mesangial cells upstream of mTOR. Cell Signal 21: 1806–1817. 19666112 10.1016/j.cellsig.2009.07.016

[46] Wang T, Donahoe PK (2004) The immunophilin FKBP12: a molecular guardian of the TGF-beta family type I receptors. Front Biosci 9: 619–631. 14766396 10.2741/1095

[47] Avila G, Lee EH, Perez CF, Allen PD, Dirksen RT (2003) FKBP12 binding to RyR1 modulates excitation-contraction coupling in mouse skeletal myotubes. J Biol Chem 278: 22600–22608. 12704193 10.1074/jbc.M205866200

[48] Brown EJ, Beal PA, Keith CT, Chen J, Shin TB, et al (1995) Control of p70 s6 kinase by kinase activity of FRAP in vivo. Nature 377: 441–446. 7566123 10.1038/377441a0

[49] Lorenz MC, Heitman J (1995) TOR mutations confer rapamycin resistance by preventing interaction with FKBP12-rapamycin. J Biol Chem 270: 27531–27537. 7499212 10.1074/jbc.270.46.27531

[50] Goodman CA, Mabrey DM, Frey JW, Miu MH, Schmidt EK, et al (2011) Novel insights into the regulation of skeletal muscle protein synthesis as revealed by a new nonradioactive in vivo technique. FASEB J 25: 1028–1039. 21148113 10.1096/fj.10-168799 PMC3042844

[51] Anjum R, Blenis J (2008) The RSK family of kinases: emerging roles in cellular signalling. Nat Rev Mol Cell Biol 9: 747–758. 18813292 10.1038/nrm2509

[52] Hou J, Lam F, Proud C, Wang S (2012) Targeting Mnks for cancer therapy. Oncotarget 3: 118–131. 22392765 10.18632/oncotarget.453 PMC3326643

[53] Vilella-Bach M, Nuzzi P, Fang Y, Chen J (1999) The FKBP12-rapamycin-binding domain is required for FKBP12-rapamycin-associated protein kinase activity and G1 progression. J Biol Chem 274: 4266–4272. 9933627 10.1074/jbc.274.7.4266

[54] Martineau LC, Gardiner PF (2001) Insight into skeletal muscle mechanotransduction: MAPK activation is quantitatively related to tension. J Appl Physiol 91: 693–702. 11457783 10.1152/jappl.2001.91.2.693

[55] Hornberger TA (2011) Mechanotransduction and the regulation of mTORC1 signaling in skeletal muscle. Int J Biochem Cell Biol 43: 1267–1276. 21621634 10.1016/j.biocel.2011.05.007 PMC3146557

[56] Spangenburg EE, McBride TA (2006) Inhibition of stretch-activated channels during eccentric muscle contraction attenuates p70S6K activation. J Appl Physiol 100: 129–135. 16179399 10.1152/japplphysiol.00619.2005

[57] Hornberger TA, Chien S (2006) Mechanical stimuli and nutrients regulate rapamycin-sensitive signaling through distinct mechanisms in skeletal muscle. J Cell Biochem 97: 1207–1216. 16315321 10.1002/jcb.20671

[58] Yip CK, Murata K, Walz T, Sabatini DM, Kang SA (2010) Structure of the human mTOR complex I and its implications for rapamycin inhibition. Mol Cell 38: 768–774. 20542007 10.1016/j.molcel.2010.05.017 PMC2887672

[59] Sancak Y, Thoreen CC, Peterson TR, Lindquist RA, Kang SA, et al (2007) PRAS40 is an insulin-regulated inhibitor of the mTORC1 protein kinase. Mol Cell 25: 903–915. 17386266 10.1016/j.molcel.2007.03.003

[60] Yoon MS, Du G, Backer JM, Frohman MA, Chen J (2011) Class III PI-3-kinase activates phospholipase D in an amino acid-sensing mTORC1 pathway. J Cell Biol 195: 435–447. 22024166 10.1083/jcb.201107033 PMC3206351

[61] Yonezawa K, Yoshino KI, Tokunaga C, Hara K (2004) Kinase activities associated with mTOR. Curr Top Microbiol Immunol 279: 271–282. 14560963 10.1007/978-3-642-18930-2_16

[62] Thoreen CC, Chantranupong L, Keys HR, Wang T, Gray NS, et al (2012) A unifying model for mTORC1-mediated regulation of mRNA translation. Nature 485: 109–113. 22552098 10.1038/nature11083 PMC3347774

[63] Gingras AC, Raught B, Gygi SP, Niedzwiecka A, Miron M, et al (2001) Hierarchical phosphorylation of the translation inhibitor 4E-BP1. Genes Dev 15: 2852–2864. 11691836 10.1101/gad.912401 PMC312813

[64] Zhang D, Contu R, Latronico MV, Zhang J, Rizzi R, et al (2010) MTORC1 regulates cardiac function and myocyte survival through 4E-BP1 inhibition in mice. J Clin Invest 120: 2805–2816. 20644257 10.1172/JCI43008 PMC2912201

[65] Walsh D, Mohr I (2004) Phosphorylation of eIF4E by Mnk-1 enhances HSV-1 translation and replication in quiescent cells. Genes Dev 18: 660–672. 15075293 10.1101/gad.1185304 PMC387241

