# Supplementary figures and images for: Mechanical Stimulation Induces mTOR Signaling via an ERK-Independent Mechanism: Implications for a Direct Activation of mTOR by Phosphatidic Acid
Source: PLoS One. 2012 Oct 15;7(10):e47258. doi: 10.1371/journal.pone.0047258 (PMC3471816; doi:10.1371/journal.pone.0047258)

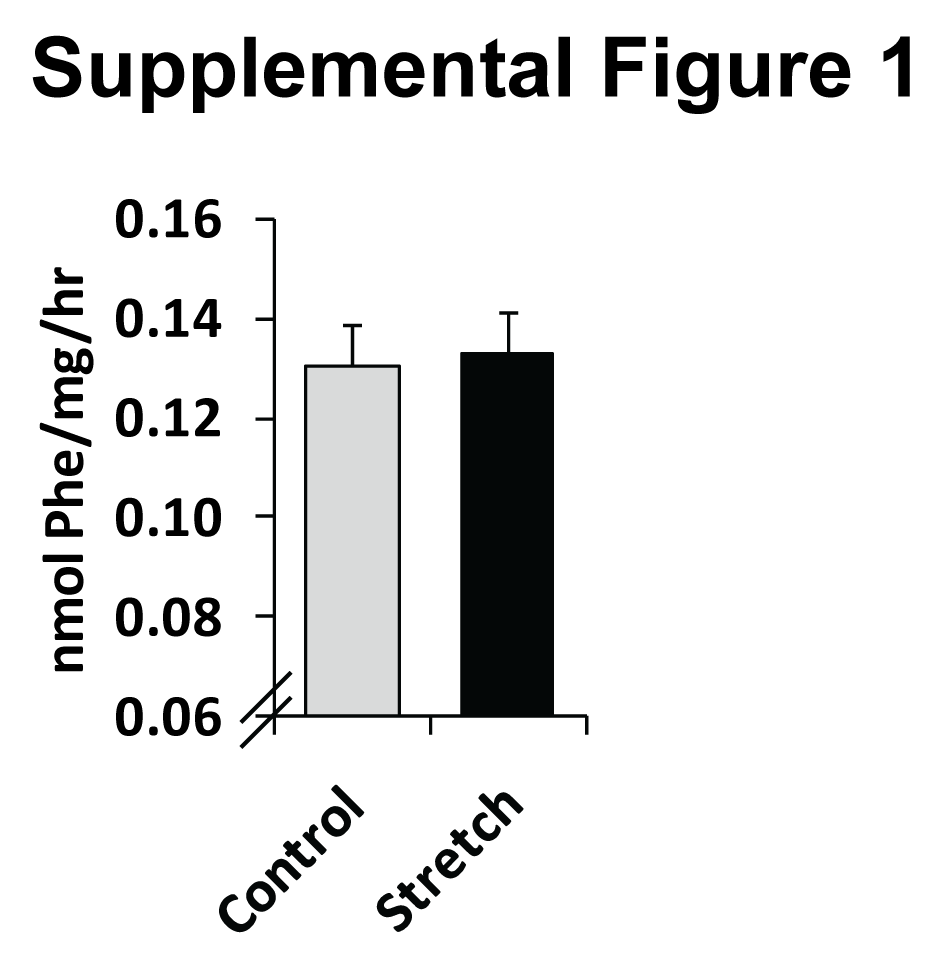

Supplement: Figure S1 — Mechanical stimulation does not increase protein synthesis in muscles incubated with KHB media. EDL muscles were held at Lo and pre-incubated for 30 min with KHB media containing 0.1% DMSO, 1X MEM amino acids and 25 mM glucose. The muscles were then subjected to 90 min of stretch or control conditions, and protein synthesis rates were measured during the final 30 min. All values are presented as the mean + SEM (n = 4−6 per group). (TIF) [file pone.0047258.s001.tif]

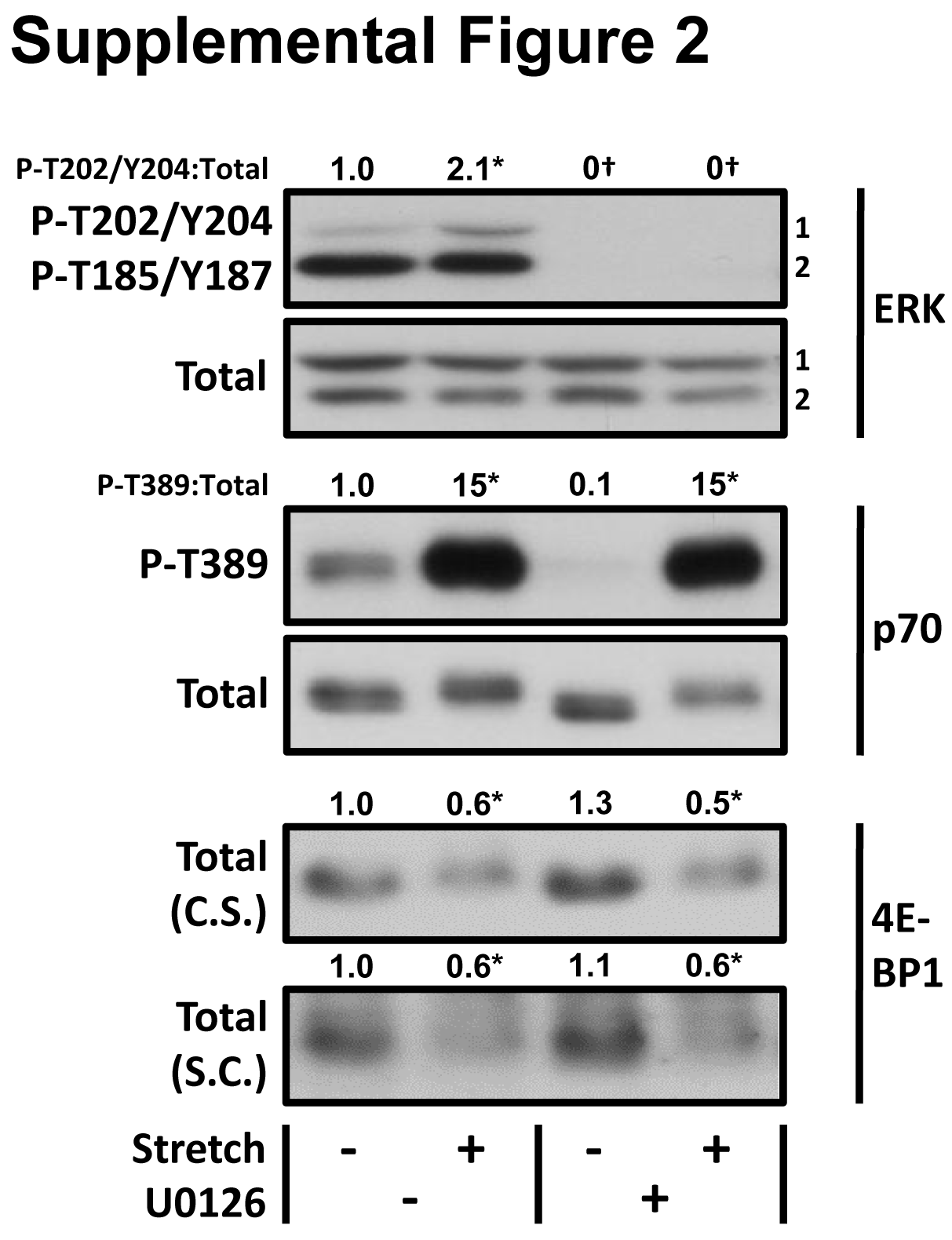

Supplement: Figure S2 — Mechanical stimulation activates mTOR signaling via an ERK-independent mechanism in DMEM media. EDL muscles were held at Lo and pre-incubated with DMEM media containing 50 µM U0126 (U0126+) or the vehicle (U0126 –, DMSO) for 30 min. The muscles were then subjected to 90 min of stretch or control conditions. Muscles were collected at the end of the 90 min interval and subjected to western blot analysis for phosphorylated (P) and total ERK, p70 and total 4EBP1. The total amount, and phospho:total ratios, of each protein were measured and then expressed relative to the values obtained in the vehicle control samples (U0126 –, Stretch –). Note: total 4E-BP1 was measured with two different antibodies, i) a monoclonal antibody from Cell Signaling that was raised against a peptide surrounding the Ser112 residues (C.S.), and ii) a polyclonal antibody from Santa Cruz that was raised against full length 4E-BP1 as the antigenic peptide (S.C.). All values are presented as the mean (n = 3−5 per group). *Significantly different from the drug-matched control group, †Significantly different from the stimulation-matched vehicle group, P≤0.05. (TIF) [file pone.0047258.s002.tif]

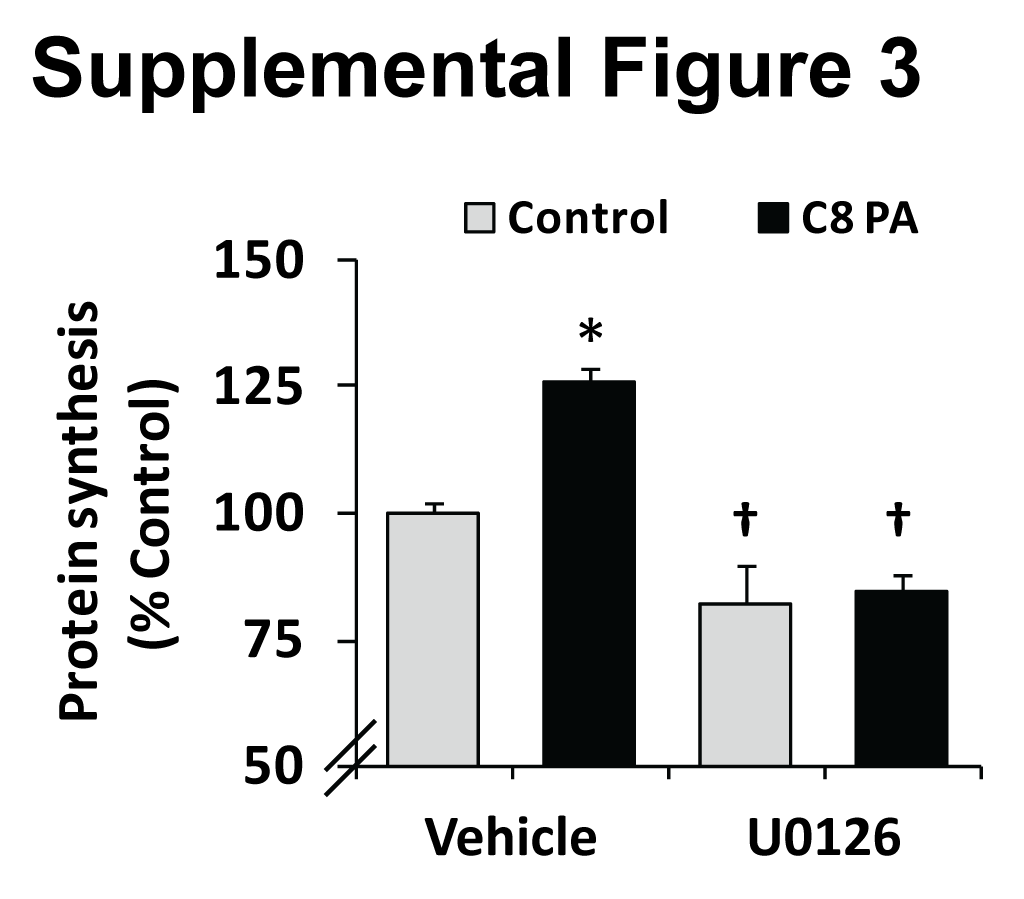

Supplement: Figure S3 — Exogenous phosphatidic acid induces protein synthesis via an ERK-dependent mechanism. C2C12 myoblasts were serum-starved overnight and then pre-incubated with 50 µM U0126 or the vehicle (DMSO) for 30 min, followed by 60 min stimulation with 30 µM C8 PA or the vehicle (PBS). Protein synthesis rates were measured during the final 30 min and expressed as a percentage of the vehicle control values. All values are presented as the mean + SEM and were obtained from five independent experiments (n = 9−11 per group). *Significantly different from the drug-matched control group. †Significantly different from the stimulation-matched vehicle group, P≤0.05. (TIF) [file pone.0047258.s003.tif]
